# Towards gender parity: An analysis of leadership management teams in Tanzania’s health sector

**DOI:** 10.1371/journal.pgph.0006813

**Published:** 2026-09-29

**Authors:** Henry Mollel, Sara Dada, Anosisye Kesale, Brynne Gilmore, Eilish McAuliffe, Magreth Thadei Mwakilasa, Ntuli Kapologwe, Ziada Sellah, Agnes Mpinga

**Affiliations:** 1 Mzumbe University - Centre of Excellence in Health Monitoring and Evaluation, Morogoro, Tanzania; 2 UCD Centre for Interdisciplinary Research, Education and Innovation in Health Systems, School of Nursing, Midwifery and Health Systems, University College Dublin, Dublin, Ireland; 3 School of Nursing, Midwifery and Health Systems, University College Dublin, Dublin, Ireland; 4 School of Public Health, Physiotherapy and Sports Science, University College Dublin, Dublin, Ireland; 5 Department of Psychiatry and Mental Health, University of Cape Town, Cape Town, South Africa; 6 East Central and Southern Africa Health Community, Arusha, Tanzania; 7 Ministry of Health, Community Development, Gender, Elderly and Children, Dodoma, Tanzania; University of Washington Department of Global Health, UNITED STATES OF AMERICA

## Abstract

Women constitute the majority of the health workforce globally and in Tanzania, yet remain underrepresented in leadership roles, particularly at higher levels. Despite national commitments to gender equality and various supportive policies, analysis of gender representation in health sector leadership in Tanzania is limited. This study presents the analysis of gender representation in leadership across three healthcare levels of Tanzania’s health system, Regional Health Management Teams (RHMTs), Council Health Management Teams (CHMTs), and Health Facility Management Teams (HFMTs). This study used secondary data collected by a government ministry in 2022 from 20 out of 26 regions of mainland Tanzania, representing over two-thirds of the country’s regions. The analysis examined 9,120 leadership positions. Descriptive statistics were done using Excel. Results show that while gender representation is nearing parity overall for less powerful roles, notable disparities remain based on role and level of seniority. The highest proportion of women is found in HFMTs at 48.9%, followed by a decline at the CHMTs level to 41.5%, and further to 39.5% in the RHMTs. This trend is similarly evident in leadership roles such as Medical Officers In-Charge, where women represent 29.8% at the HFMT level, but their presence drops to 17.6% among District Medical Officers and just 11.1% among Regional Medical Officers. Gender representation also varies geographically and by facility type, with lower-level facilities generally showing more equitable gender distributions. The findings reflect the ongoing need for gender-transformative leadership strategies, including targeted mentorship, equitable promotion practices, and inclusive workplace cultures.

## Introduction

Women show a predominance in the health sector worldwide, yet their opportunities to influence decision-making remain limited [1]. For instance, in Spanish public hospitals, it was found that a man had 3.85 times the probability of acquiring a management position than a woman, and that the representation of women in leadership is lowest in positions with greater responsibility [2]. In Zambia, Women in Global Health surveyed 99 districts and provinces and found that only 13% of districts and 10% of provinces had female leadership representation [3]. In Mozambique, studies have highlighted that women constitute approximately 40% of physicians and 56% of nurses and midwives; despite this, they are underrepresented in decision making roles and they face significant barriers to career advancement [4]. In Central Malawi, women face challenges in advancing to decision-making roles, as they are less likely than men to secure higher responsibility positions in the health system [5]. Similar experiences were reported in India, Ethiopia, Kenya and Pakistan, where the proportion of women in leadership positions in the health sector are low, despite women being the majority in the health workforce [6–8]. This inequitable leadership also pertains to governing bodies related to global health decision-making. Between 1948 and 2021, 82.9% of delegates at the World Health Assembly were men, with less than 30% of Chief Delegates being women [9].

The recent Lancet Commission on gender and global health recognises gendered inequalities in the health workforce and health governance as critical in achieving gender justice in global health worldwide [10]. Women in leadership positions in healthcare drive an equitable and supportive environment for women, children and marginalised populations [11]. Furthermore, women in leadership are proactive in strengthening health service delivery tailored to the community’s needs and preferences, improving health outcomes for the population [12]. A recent global scoping review of 137 articles found that women in health leadership roles positively influenced financial performance, innovation, ethical initiatives, health, organisational culture, and other women’s careers [13].

Despite this, progress in realising equal opportunities for women in decision-making and leadership, particularly in senior healthcare positions, is slow [14]. There is limited data from many countries on the breakdown of gender leadership in the health workforce and on the impacts of inequitable gendered leadership. Barriers to women’s leadership in healthcare can be multifaceted. A recent study from East Africa identified gender stereotypes, male-dominated cultures, workplace dynamics, and family and caregiving responsibilities as key barriers that affect women’s leadership trajectories [15]. Within Tanzania specifically, a lack of confidence and self-efficacy, institutional bias and gatekeeping, as well as gender stereotypes and an underlying misinterpretation of women’s leadership styles, were reported to influence women’s leadership in healthcare [16]. Additionally, overload due to family responsibilities, cultural expectations and systemic barriers are also impeding women’s advancement to leadership positions [16]. Worldwide there is limited analysis of the gender breakdown in healthcare leadership. Without further evidence on the gender breakdown in healthcare leadership, planning for and investing in advancing women to powerful and senior leadership positions will remain challenging.

In Tanzania, the National Policy on Women Development and Gender (NPWDG) provides a framework for gender equality and women’s empowerment [17]. The National Strategy for Gender and Development (NSGD) supports the operationalisation of the NPWDG, highlighting interventions that need to be implemented and the responsible actors [17, 18]. It calls for mainstreaming gender issues across policies, plans, strategies, programs, budgets, and activities to bridge the existing gender gaps in all sectors [18,19]. Additionally, the NPWDG approved the National Sub-Program on Women and Gender Advancement in 2000, which implemented four of the priority areas identified in the Beijing Platform for Action: 1) legal capacity, 2) education, 3) political empowerment, and 4) economic empowerment^17^. Despite these policy frameworks, evidence of the impact on gender representation is mixed. A 2024 survey of civil servants in Tanzania found significant barriers to gender equality in governance, such as the lack of gender-sensitive policy implementation and persistent institutional biases [20]. Efforts to close these gaps in representation include structural mechanisms such as gender focal points in government bodies and gender-responsive budgeting, though evidence on their influence on gender-equitable resource allocation remains limitted [21]. Other approaches include interventions focused on capacity building, mentorship, and training for aspiring women leaders (such as the UONGOZI Institute’s Women’s Leadership Programme) [22].

Despite the numerous efforts by Tanzania to reduce gender inequality and promote women’s involvement in leadership structures throughout the workforce, there is currently no analysis of data on gender representation in management teams which are leadership structures across the health sector. Without information on who holds powerful and senior leadership and decision-making positions, measuring progress in leadership equality or designing appropriate strategies to improve existing inequalities is difficult. This study analyses and presents the available data on gender in the Tanzania health sector leadership structures in order to highlight areas where there is need for action to achieve gender equality.

### Tanzania’s health system

The Tanzanian health system is decentralised cascading from tertiary to primary levels, involving multiple levels of management to ensure effective planning, coordination, supervision, and implementation of health services. Fig 1 presents a visual representation of the structures and levels in the Tanzanian health system [23]. The Regional Health Management team (RHMT) and Council Health Management team (CHMT) are leadership structures at regional and council levels - responsible for secondary and primary health services, respectively. At the health facility level, the responsibility lies with the Health Facility Management Team (HFMT). By design, the membership of RHMT, CHMT and HFMT includes coordinators in respective administrative levels, heads of relevant departments, sections and units, with a small number of co-opted members brought in for specific purposes and time. This study analyses the gender representation in leadership and membership of the RHMTs, CHMTs, and HFMTs. In doing so, this study examines whether gender inequality is greater at the higher levels of leadership structures and maps geographical differences.

**Fig 1:**
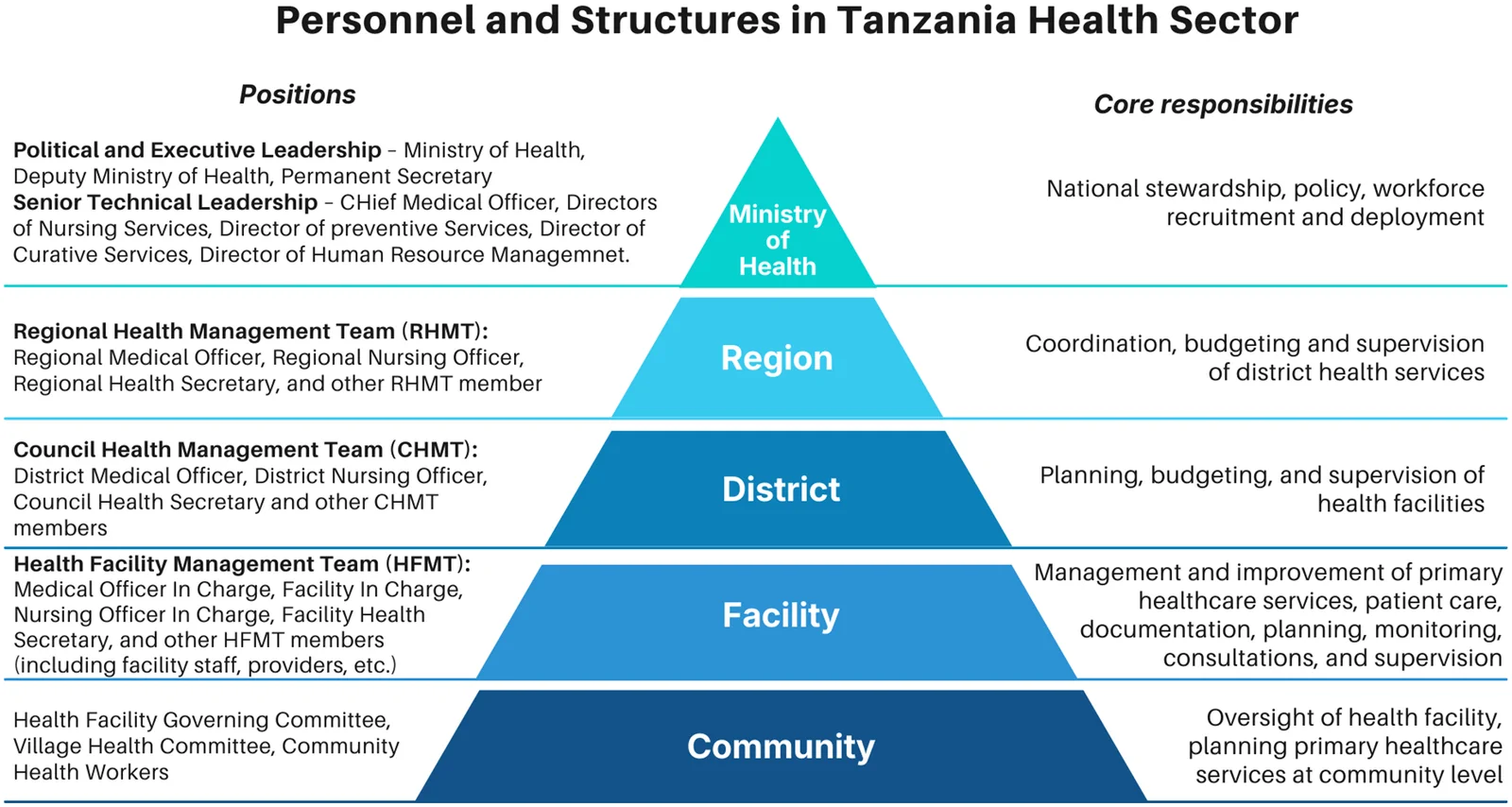
Tanzanian health system.

## Methods

This cross-sectional study used secondary data supplied by the Ministry in Tanzania upon written request in 2022. This data is part of routine data collection conducted quarterly through regional, district and facility leaders. The initial purpose of reviewing this data was to help identify sites for the implementation of the Gendered Leadership, Advancing Systems and Structures (GLASS) project [17]. The GLASS project is an implementation research project that aims to address the organisational and individual barriers to the advancement of women in health leadership positions in Tanzania [17].

### Ethics statement

This research is part of the GLASS project, which received ethical approval number (NIMR/HQ/R.8a/Vol.IX/4282)obtained from the National Institute of Medical Research (NIMR) in Tanzania. It has also received ethical approval from Mzumbe University and University College Dublin (LS-C-22–220-Mucheru).

### Data collection/sourcing

This was an anonymised dataset. Raw data listing titles and genders of members of the RHMT, CHMT, and HFMT were collected electronically by the Directorate of Nursing and Midwifery through Regional Nursing Officers (RNOs) and District Nursing Officers (DNOs) between March and June 2022. The available data were compiled into three lists—RHMT, CHMT, and HFMT—and cleaned for consistency in nomenclature.

Gender data was included in the source data as ‘Man,’ ‘Woman,’ or ‘Unknown.’ Where the gender data field was blank, it was considered ‘Unknown.’ For the RHMT, four categories of roles were included: Regional Medical Officer (RMO), Regional Nursing Officer (RNO), Health Secretary, and Other. Similarly, the CHMT dataset included: District Medical Officer (DMO), District Nursing Officer (DNO), Health Secretary, and Other. For both RHMT and CHMT, these roles were explicitly stated in the dataset. For the HFMT, five categories of roles were included: Medical Officer In-Charge (MOI), Health Facility In-Charge, Nursing Officer In-Charge (NOI), Health Secretary, Section/Unit In-Charge, Healthcare Provider, Administrative In-Charge, Administrative Support, and Other. Table A in S1 Appendix demonstrates how the original phrasing of roles in the dataset was categorised into these eight categories.

For the HFMT dataset, facility types were determined based on the facility’s name as provided in the dataset and/or the MOH Health Facility Registry (https://hfrs.moh.go.tz/web/index.php). Dispensaries, health centres, district-level hospitals and regional-level hospitals were included. Three facilities (one private medical clinic, one national super-specialised hospital, and one unknown facility) were excluded because they did not match the above categories, which were based on the Tanzania MOH Health Facility Registry.

### Data analysis

Descriptive statistics were calculated for the gender representation in membership of RHMTs, CHMTs and HFMTs as well as leadership positions within these teams (e.g., regional/district medical officer, health secretary, health facility in-charge). In this analysis, gender parity refers to 45–55% representation of women in that role or team. Visual representations of the data and charts were developed using Excel and R.

## Results

Data from 20 of Tanzania’s 26 regions of mainland Tanzania was provided (demonstrated in Fig 2). A total of 9,120 positions and their genders were reported. In total, the dataset included 4,231 women, 4,778 men and 101 individuals with unknown genders.

**Fig 2:**
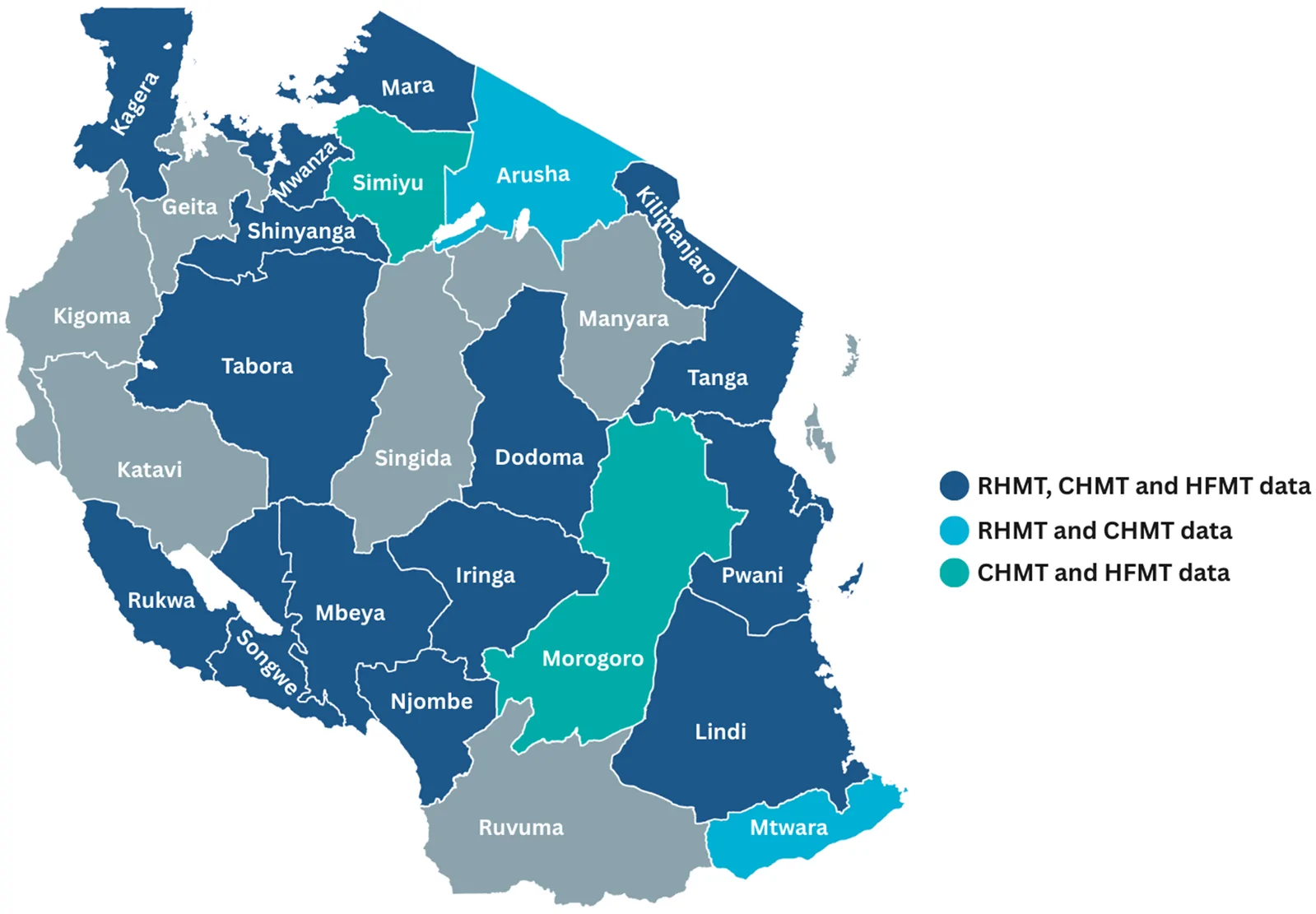
Regions of Tanzania included in the dataset (created using Paintmaps.com).

Final RHMT data included 370 individuals from 18 regional teams (Table B in S1 Appendix). Final CHMT data included 2620 individuals from 125 councils across 20 regions (Table C in S1 Appendix). Final HFMT data included 6130 individuals from 1793 dispensaries, 263 health centres, 43 district hospitals and 6 regional referral hospitals across 18 regions (Table D in S1 Appendix). Table 1 summarises the proportion of women in roles across the regional, council and health-facility levels in Tanzania’s health system. Detailed breakdowns of each health management team by region are presented in Tables B-D in S1 Appendix.

**Table 1:** Summary of gender representation in regional, council and health facility management teams in Tanzania.

Gender Representation in Health Management Team Roles	
	**Women % (women/total members)**
**Regional Health Management Teams (RHMTs)**	
Overall RHMT	39.5% (146/370)
Regional Medical Officer (RMO)	11.1% (2/18)
Regional Nursing Officer (RNO)	77.8% (14/18)
Health Secretary	66.7% (12/18)
Other	37.3% (118/316)
**Council Health Management Teams (CHMTs)**	
Overall CHMT	41.5% (1087/2620)
District Medical Officer (DMO)	17.6% (22/125)
District Nursing Officer (DNO)	75.4% (95/126)
Health Secretary	44.8% (56/125)
Other	40.7% (914/2244)
**Health Facility Management Teams (HFMTs)**	
Overall HFMT	48.9% (2998/6130)
Health Facility in-charge/Medical Officer In Charge (MOI)	29.8% (623/2091)
Nursing Officer In Charge (NOI)	65.9% (927/1407)
Health Secretary	63.8% (30/47)
Section/Unit In Charge	59.2% (984/1662)
Other	47.0% (434/923)

The percentage of women’s representation in leadership roles ranged from 11% to 77%. Overall, the findings demonstrate that at all levels of the health leadership teams, women occupy less than 50% of leadership roles, with a progressive decrease in gender parity at the higher levels and more powerful roles in the health system. The HFMTs have the highest proportion of women (48.9%), while this decreases at the CHMT level (41.5%) and RHMT level (39.5%). A similar pattern is reflected in the role of Health Facility In-Charge (who are medical officers), where Health Facility In-Charge at the HFMT level are 29.8% women, yet only 17.6% of District Medical Officers and 11.1% of Regional Medical Officers are women. However, 65.9% of Nursing Officers-In Charge at the HFMT level are women, which increases to 75.4% of District Nursing Officers and 77.8% of Regional Nursing Officers being women. The role of health secretary at all levels of the health management teams is consistently at parity or a majority of women (63.8% at the HFMT level, 44.8% at CHMT and 66.7% at RHMT).

### Zone

Fig 3 demonstrates mixed results of gender representation across the eight zones. Across zones and health management team levels, women’s representation never exceeds 60% (Northern zone’s HFMTs). No zone has a majority of women in all three health management team levels, with only the Central and Northern zones’ RHMTs reaching parity. The Western and Southern zones have notably low women representation in the RHMTs, with 30% and 32% respectively. These proportions increase slightly at the CHMT and HFMT levels in both zones, yet remain below 50%. At the CHMT level, only the Eastern and Western zones approach gender parity in their leadership structures representation. The Southwest Highlands zone shows fairly low representation of women (37–40% across all three levels). Meanwhile, the Eastern and Lake zones hover around gender parity at the HFMT level, yet this drops at the CHMT level (for example, 56% HFMT vs. 48% CHMT in Eastern). Across zones, HFMTs typically have higher proportions of women representation (ranging from 40-60%).

**Fig 3:**
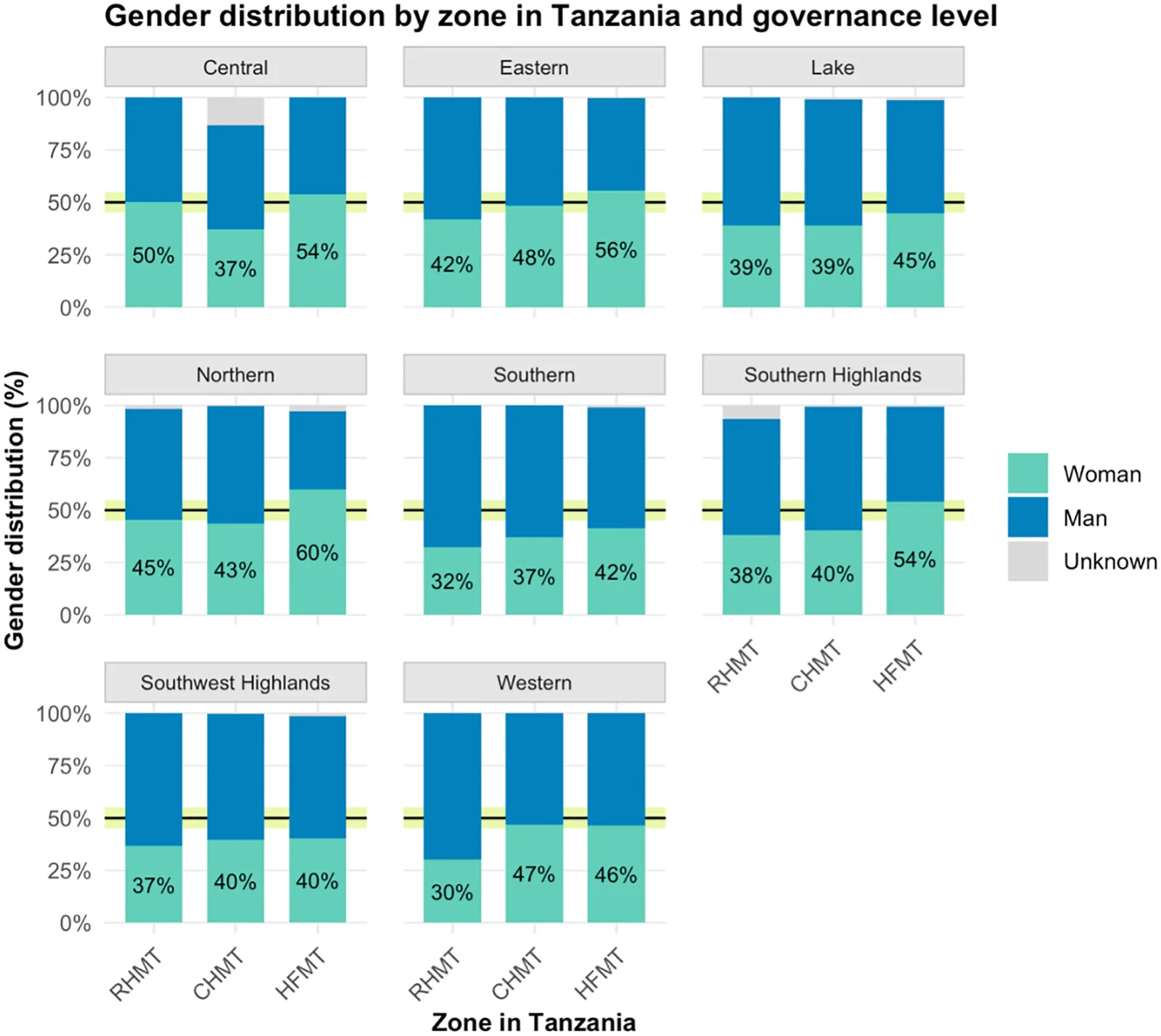
Gender distribution by zone in Tanzania and governance level.

### Region

Fig 4 presents a visual representation of the RHMT, CHMT and HFMT by region. Based on this data, the percentage of women in leadership structures is typically higher at the lower levels, with the percentage declining at the higher levels of the health system. This is true for 14 of the 20 regions, demonstrated in Fig 4. Defining gender parity as 45–55% (indicated by the highlighted line in Fig 4), four of the 18 RHMTs, six of the 20 regions’ CHMTs, and seven of the 20 regions’ HFMTs demonstrate parity in their teams. At the RHMT level, Arusha (55%) and Mbeya (56%) are the only two regions with a majority of women in their leadership team. At the CHMT level, Dar Es Salaam has a proportion of 59% women. The highest representation of women on leadership teams happens at the HFMT level, with Kilimanjaro (61%), Dar Es Salaam (58%), Iringa (56%) and Pwani (56%) all demonstrating a majority (over 55%) of women in their overall leadership team’s membership.

**Fig 4:**
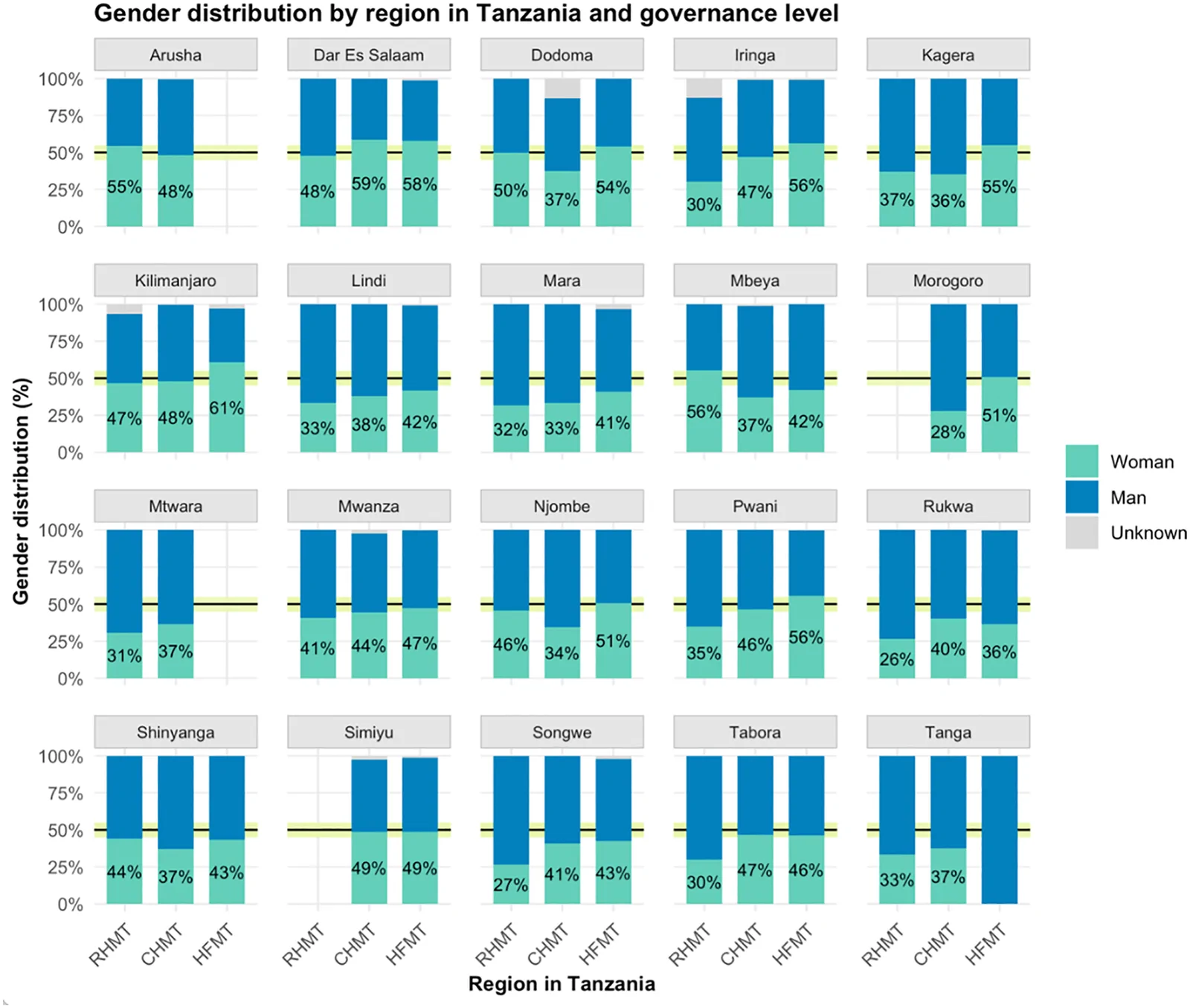
Gender distribution by region in Tanzania and governance level.

### Health facility types

The HFMTs at the facility level were also further analysed by facility type, presented in Fig 5. These findings demonstrate parity at the lower-level facilities: dispensaries (48% women), health centres (50%) and district/district-level hospitals (50%). However, the proportion of women in these structures decreases at the regional-level hospitals to 44%.

**Fig 5:**
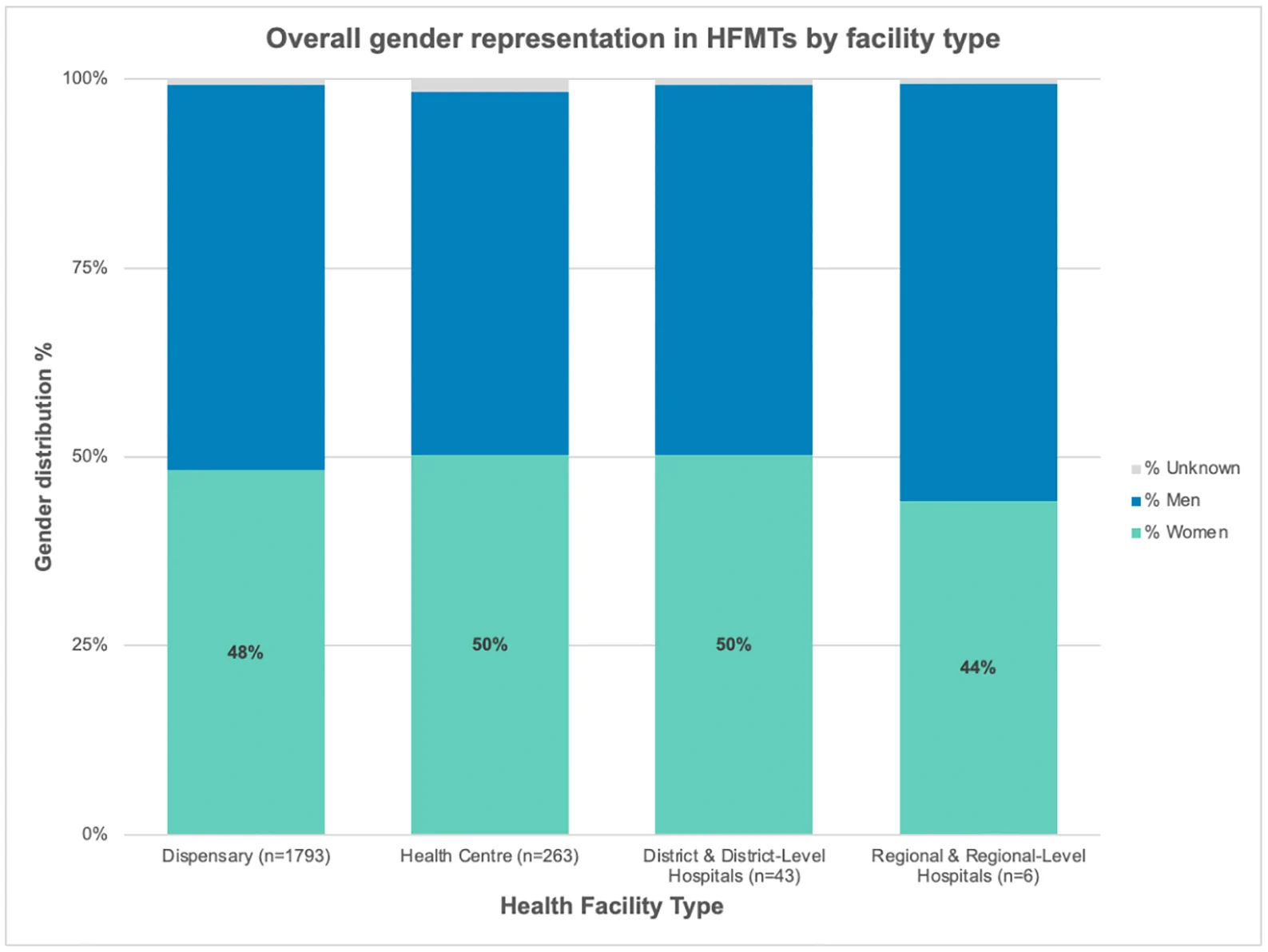
Overall gender representation in HFMTs by facility type.

Additionally, Table 2 provides a more detailed breakdown of the proportions of women in different roles in the HFMTs.

**Table 2:** Representation of women in different HFMT roles by facility type.

	Dispensary (n = 1793)	Health Centre (n = 263)	District & District-Level Hospitals (n = 43)	Regional & Regional-Level Hospitals (n = 6)
**Health Facility In-Charge/MOI/DMO/RMO**	555	54	14	0
	***32.6%***	***19.6%***	***13.6%***	***0.0%***
**NOI**	677	175	68	7
	***63.6%***	***72.6%***	***73.1%***	***77.8%***
**Health Secretary**	0	10	13	7
	***0.0%***	***71.4%***	***52.0%***	***87.5%***
**Section/Unit In- Charge**	375	349	250	10
	***65.7%***	***56.2%***	***58.7%***	***22.7%***
**Other**	159	113	122	40
	***48.9%***	***46.7%***	***43.4%***	***53.3%***

### Professional roles

Examining the differences across roles and professional groupings, the proportions of women are high in Nursing and Health Secretary. The representation of women in Medical Officer In-Charge roles decreases at higher levels of health facilities and in the overall health system. For example, 32.6% of MOIs at the dispensary level are women, this decreases to 19.6% at the health centres, 13.6% at the district hospitals and 0% in regional hospitals (Table 2). Similarly, while the overall percentage of women MOIs in HFMTs is 29.8%, only 17.6% of DMOs are women at the CHMT level, and this drops further to 11.1% of RMOs in the RHMTs. Examining the representation at the HFMTs closer, we see that the proportion of women healthcare providers ranges from 46-60% across the different facility types. While at the dispensary level, 65.7% in-charges are women, this proportion drops to 59.2% in district hospitals and 56.1% in health centres. At the regional hospital level, 60% of healthcare providers are women, but only 20% of in-charges are women.

## Discussion

Worldwide, women comprise the majority of healthcare workforce, yet they are underrepresented in leadership positions. However, statistics on the gender breakdown of healthcare leadership at the country level are limited. This study addresses this gap for Tanzania by providing a snapshot on women representation in leadership structures. Findings reveal that while gender representation is impressively strong at the HFMT level, it is weaker at the higher levels of the health system (the CHMT and RHMT levels). Overall, women’s representation in leadership structures in Tanzania’s health system are approaching parity. However, this trend is largely driven by women’s greater presence in what may be considered lower-power positions and less-specialised roles, such as coordination and secretarial positions. In contrast, women are notably absent from the more influential or powerful roles, such as DMOs and RMOs. The individuals occupying these positions frequently serve as the chairpersons of these leadership management teams, meaning they play a key role in final decisions. While the pattern of declining representation of women at higher levels of the health system has been consistently documented globally [24], this study makes a distinct contribution by providing granular, system-wide data across three tiers of primary healthcare in Tanzania. Crucially, it moves beyond documenting overall representation to revealing how women’s presence is concentrated in lower-power and less-specialised roles, even where headline parity figures appear encouraging, offering a more nuanced picture of gendered leadership structures than aggregate statistics alone can provide.

Leadership structures in the healthcare industry are usually filled by appointment in Tanzania, rather than an open application process. Senior leaders select candidates whom they consider most suitable, based on their job performance and education level. Under frameworks such as NPWDG and NSDG, those making these appointments are expected to give equal consideration to women and men for roles. However, as the evidence above indicates, this expectation may not yet be translated into proportionate representation in practice. In her study of women participation in leadership in the Tanzanian government, Makulilo argues that the “*constructed social reality that women are not supposed to lead (voiced in her data as ‘cannot make difficult decisions’) and are weak (voiced as ‘depend on favours’) contributes much in limiting women representation in top managerial positions*” [25]. This is further compounded by weak institutional frameworks that hinder the promotion of gender equality in decision-making [26]. This may, in part, explain why women are being overlooked for these top positions.

Organisations often reflect the culture of the societies in which they are placed [27]. Societies often vary in their approach to gender roles, with some working to actively reduce differences while others may not. These tendencies are closely tied to broader questions of gender stereotyping, role ideology, and discrimination, as deeply held societal beliefs about what men and women are capable of and/or expected to do, inevitably shape how roles and responsibilities are distributed between them [16]. Such entrenched biases and stereotypes can create an environment in which women may unconsciously find it harder to see themselves as leaders [28,29]. Acker’s theory of gendered organisations [30] argues that organisational hierarchies are not neutral but are built around implicit masculine norms, rendering them structurally disadvantageous to women — particularly at senior levels. This helps explain why, even where gender-sensitive policies exist, women remain concentrated in lower-power roles. Addressing this requires more than individual-level change; it calls for systemic intervention to foster genuine recognition of women’s leadership potential [31].

The pattern observed in Tanzania’s health system (strong representation at facility level, declining sharply toward regional and district leadership) is also consistent with what scholars describe as the “leadership pipeline” or “leaky pipeline” effect, whereby cumulative structural and cultural disadvantages reduce women’s progression at each successive level [32]. Vertical occupational segregation further compounds this: women dominate the health workforce numerically, especially in the nursing cadre, but are systematically excluded from the roles that carry decision-making authority [24,33]. In Tanzania, the positions (such as Regional Medical Officers, District Medical Officers and Medical Officers In Charge), which are majority men, have the power to influence decision-making, planning, and resource allocation at different levels of the health system, thereby influencing the interpretation and implementation of interventions focused on addressing gender disparities.

These findings demonstrate that while women constitute the majority in nursing-related leadership positions, largely reflecting their predominance in these cadres; however, this has not translated into broader representation in more influential leadership roles, even at the facility level where overall gender parity appears closer. This may partly explain why the data shows regional variation, but less variation by facility type. Leadership positions at all facility levels within a region are decided by the RHMT and gender equity across the region is therefore dependent on the attitudes of these RHMT members to women leaders. These findings, along with the broader literature on gendered organisations and the health workforce [9,11,24,30,32,33], suggest that the underrepresentation of women in DMO and RMO positions in Tanzania is not incidental, but reflects broader structural patterns that require deliberate systemic intervention. Weak gender-sensitive policies and inadequate support systems can limit women’s ability to take on leadership roles, further influencing the effectiveness of existing frameworks aimed at promoting gender equity in health leadership [26].

This mirrors global evidence from low- and middle-income countries. Women are concentrated in lower-paid, lower-status positions, while men disproportionately occupy key leadership roles with decision-making authority. This persistent stratification highlights the structural inequities embedded within health systems [34]. This pattern suggests that while women may be present in the workforce, structural and cultural barriers continue to impede their progression into senior leadership roles [8]. Many writers have suggested that men and women managers view leadership roles differently, with men viewing hierarchical and competitive behaviours as components of leadership while women prioritise collaboration and consensus-building [16,35]. Perceptions of gender differences in leadership characteristics take on particular significance in a system where career advancement decisions are made by regional health committees, which tend to be more dominated by men. This gender imbalance may contribute to a bias in favour of appointing men to leadership positions. One explanation for this may be that societal beliefs about what men and women ‘can’ and ‘should’ do can reinforce preferences for allocating roles along ‘traditional’ gender lines [36]. Similar patterns are noted across multiple settings, highlighting how the “leaky pipeline” in leadership trajectories reflects cumulative disadvantage and gendered norms that discourage or hinder women’s advancement [8,37].

These patterns reinforce traditional gender roles in the health sector and may limit the visibility and aspirations of individuals in underrepresented groups. Research indicates that visible role models in leadership positions can significantly influence career aspirations and retention, particularly among women and marginalised groups [38]. Increasing the visibility of women in medical leadership roles may help address the gender imbalance in medical professions, just as more men in nursing leadership might challenge persistent gender stereotypes in caregiving professions. This approach aligns with findings from the BMJ Gender Equality in the Health Workforce collection [39], which emphasises that gender-transformative leadership is essential to dismantle stereotypes and promote inclusive leadership structures that reflect the diversity of the health workforce.

However, merely increasing representation without structural change does not address existing power imbalances [40,41]. Women in healthcare leadership often face systemic barriers, including unsupportive work environments, a lack of confidence, and challenges balancing family and work responsibilities [6]. These obstacles are frequently rooted in patriarchal cultural norms that assign conventional roles and expectations to women [6,42,43]. Addressing these challenges requires more than reflecting the current gender composition in various professions; it necessitates proactive strategies to support diverse populations pursuing and thriving in these careers [37, 44]. Creating targeted mentorship programmes, implementing equitable promotion criteria, and fostering organisational cultures that value diverse leadership can contribute to more balanced representation [37]. A transformative approach to leadership development in the health sector is crucial not only to achieving gender equity but also to improving health outcomes through more inclusive and responsive governance.

### Implications for policy, practice, and research

Tanzania has made substantial progress in supporting the SDGs and working towards more gender equitable societies and services. There is a clear commitment to prioritising gender and women’s empowerment, as demonstrated by the NPWDG and the NSGD [17]. Tanzania’s decentralised health system means that there are opportunities for improved representation in administrative roles and consequently leadership structures [45]. This study demonstrates that progress towards gender parity is occurring in coordinating and administrative roles, whilst presenting clear evidence of the need for policies and strategies that support the continuation of parity into the more influential leadership roles. In order to benefit from women’s involvement in leadership and decision making at more senior levels, there is a need to examine policies that may be hindering this, for example confining eligibility for certain senior leadership positions to professions that are dominated by men. The findings of this study also point to the need to review promotion policy and practice to ensure that women are not disadvantaged by gender bias and gendered perceptions of effective leadership attributes. Continued monitoring of promotion statistics disaggregated for gender is critical to detecting and monitoring such biases. Training in unconscious bias may be necessary where there is evidence of gender inequality.

Key recommendations to advance women’s leadership have included: identifying the training resources that target the challenges women face on an individual level; creating more inclusive cultures and processes at the organisational level; promoting gender transformative policies; working collaboratively with partners to scale impact; and contextualising solutions for local environments [46]. Formal mentorship and leadership pipeline programmes have been often recommended and demonstrate promise. For example, WomenLift Health’s 12-month Leadership Journey, operating across East Africa, brings together mid-career women in health through mentorship, coaching, and peer networks. The GLASS project is another example of such a programme that has been co-designed and implemented in Tanzania [17]. Setting gender parity targets for senior appointments such as DMO and RMO roles, with accountability through the gender-disaggregated monitoring already recommended, would complement these mentorship and leadership pipeline efforts. Collectively, these should be underpinned by gender-transformative approaches — defined by the WHO [24] as going beyond numerical representation to actively dismantling the norms and structures that sustain inequality, including embedding gender equity objectives into the performance frameworks of regional health management teams.

Strengthening accountability mechanisms will also be essential for translating these policies and global commitments into practice. While the NPWDG emphasises the development of indicators for measuring gender equality in national initiatives, and the NSGD calls for gender mainstreaming across all policies, programmes and budgets, neither framework currently incorporates specific indicators for tracking women’s representation in health leadership roles. Relatedly, the absence of comparative data from an earlier period means that assessing progress is not possible. Furthermore, the inconsistencies and incomplete dataset suggest the need for more robust data collection in order to monitor progress. Incorporating leadership-specific gender indicators — such as the proportion of women in DMO, RMO, and MOI positions — into existing national monitoring and evaluation frameworks would provide a more robust basis for accountability. This dataset could serve as a baseline for such monitoring in Tanzania, and these indicators could be embedded within the Ministry of Health’s existing data infrastructure and reporting cycles. Future research on this topic can also contribute to developing a longitudinal dataset or conduct time-series studies to analyse how representation changes over time. Monitoring and tracking leadership of medical and nursing officers and comparing with the gender breakdowns of the registered professionals can also indicate if the leadership breakdown is proportionate to staff gender, or if it is more based on existing stereotypes and inequality within the professions. Similar analyses of gender representation in the health system across different countries could contribute to a more comprehensive global understanding of gendered leadership in health care.

### Strengths & limitations

This work reports on an extensive area of Tanzania, encompassing two-thirds of the regions and leadership teams within Tanzania, and includes representation from all nine administrative zones. Another strength of this work is reporting across three tiers of primary healthcare in Tanzania. This work, therefore, adds to growing evidence based on gender in healthcare and addresses numerous calls for more transparency and reporting of leadership data. Working with the Ministry to access and report on this data strengthens the accuracy and relevance of this work. Relatedly, the dataset was drawn from the Ministry’s routine data collection, involving information reported directly by the facilities and teams. As such, the data are self-reported rather than inferred, which is considered a preferred approach for collecting and analysing gender-related information [41].

One limitation of this cross-sectional research is that it provides data on the gender makeup at only one time point. These roles are frequently changing, and the gender distribution may be slightly different at different time points. Additionally, data from this study came directly from the Ministry, making it difficult to fully account for potential errors in data entry and missing data. It is also important to note that the representation of data varies across the geographical areas studied. Relatedly, the available data was not nested – and not every HFMT, CHMT or RHMT within each region and at every level were available. Variations in sample sizes of the different teams could impact the comparability of findings across regions and zones. Therefore, while common themes were identified, these results should be interpreted with caution when comparing geographical areas, and further research is needed to explore the role of local factors in shaping the findings. However, this study presents a snapshot and can therefore serve as a potential initial indicator of the state of gender representation in these leadership structures.

## Conclusion

This study contributes to an increased understanding of gender representation in healthcare leadership within Tanzania. While there is encouraging evidence of progress, particularly near-parity in less influential leadership roles such as coordination and section head positions, this progress does not extend to the more influential roles such as Regional Medical Officers, District Medical Officers and Medical Officers In-Charge. These key positions, which hold substantial decision-making authority, remain predominantly occupied by men, reflecting persistent gender disparities at the higher levels of the health system. To achieve meaningful change, efforts should focus on adopting transformative leadership development approaches and strengthening institutional frameworks and mentorship structures to effectively promote and support gender-related programmes. This includes supporting women through mentorship, creating equitable promotion pathways, and cultivating inclusive organisational cultures. Additionally, this analysis provides a foundational benchmark for measuring future progress in gender equity within Tanzania’s health sector. The Tanzanian government can leverage this to assess the impact of current gender equity policies and adjust strategies accordingly to ensure more inclusive leadership development.

## Supporting information

S1 Appendix Table A: Health Facility Management Team Job Titles Categorised. Table B: Regional Facility Management Team Results. Table C: Council Health Management Team Results. Table D: Health Facility Management Team Results.
(DOCX)
