# Supplementary material for: Towards gender parity: An analysis of leadership management teams in Tanzania’s health sector
Source: PLOS Glob Public Health. 2026 Sep 29;6(9):e0006813. doi: 10.1371/journal.pgph.0006813 (PMC13623133; doi:10.1371/journal.pgph.0006813)
Supplement: Supplementary file 1 [file pgph.0006813.s001.docx]

**Appendix Materials**

**Table A. Health Facility Management Team Job Titles Categorised**

| ***Title category*** | ***Original title provided in raw data (e.g. Supervisor, Head of Section/Unit/Department)*** |
| --- | --- |
| **Admin In Charge** | Accountants Incharge, Chief Of Finance, Chief Of Hospital Services, H/C Accountant, Head Of Account And Finance, Head Of Procurement Unit, Hod Procurement Unit, Laundry Depart-Incharge, Laundry Ma Incharge, Record I/C, Recorder Incharge |
| **Admin Support** | Accountant, Accountant (EN), Accounts, Accountant, Administrator, Asst Account Officer, Biomedical Engineering, Biomedical Engineer, Dispensing, Dispenser, Finance, Financing, Hosp Accountant, Hospital Accountant, Hospital Treasurer, Hospital Biomedical Engineer, HRO, Hs/Administrator, Human Resource, Human Resource Officer, Human Resources, Internal Auditor, It, Laundry, Loundly, Procurement, Procurement And Supply Officer, Procurement Officer, Reception, State Manager, Storekeeper, Supportive Services Co, Supplies Officer, Supportive Co, Supportive Service Coordinator, Treasurer |
| **Health Secretary** | Health Secretary, District Health Secretary, HHS, Hospital Health Secretary, Hospital Secretary, HS, Secretary, Town Health Secretary |
| **Health Section In Charge** | CTC Incharge ANO, Incharge CTC, Incharge Op Theatre, Incharge Dental Department, Incharge Lab, Incharge Pharm, Incharge Pharmacy, Incharge X-Ray Department, Lw Incharge, Maty Nursing Officer Incharge, Ano I/C, Ass. Nurse Officer Rch Incharge, Assistant Nursing Officer (Labour Ward Incharge), Assistant Nursing Officer Incharge (Ctc), Assistant Nursing Officer Incharge (Rch), Assistant Nursing Officer Incharge ( Bima Ipd), Assistant Nursing Officer Incharge (Female Ward), Assistant Nursing Officer Incharge (Male Ward), Assistant Nursing Officer Incharge (Pediatric Ward), Assistant Nursing Officer Incharge (Ant-Natal), Assistant Nursing Officer Incharge (Labour Ward), Assistant Nursing Officer Incharge (Labour Ward) Vip, Assistant Nursing Officer Incharge (Labour& Rch), Cct Incharge, Cecap Co (Ano), Clinical Coordinator, Clinical Officer Rchc, Clinical Service Incharge, Clinician Opd Incharge, Clinical Officer Incharge (Opd), Co I/C, Co Incharge, Co Incharge (Ctc), Ctc Ass Nursing Officer Incharge, Ctc Incharge, Ctc I/C, Ctc Incharge, Ctc Incharge (Ano), Ctc Incharge (Co), Ctc Incharge (En), Ctc Incharge-Ca, Ctc Incharge-Co, Ctc Incharge-En, Ctc Nurse I/C, Ctc Nurse Incharge, Dacc, Data Focal, Dental I/C, Dental Incharge, Dental Manager, Dental Depart-Addo Incharge, Dental Department Incharge, Dental I/C, Dental Incharge, Dental Incharge -Dental Assist, Dental Unit I/C, Dental Unit Incharge, Dental Unity Incharge, District Dental Officer, District Health Officer, District Hpco, District Pharmacist Officer, District Social Welfare, District Lab Manager, Divo, Doctor Incharge (Oth), Doctor Inharge (Paediatric), Doctor Inharge (Surgery Ward), Dpham, Dr I/C Labour Ward, Dr Incharge Maternity, Dr Incharge Opd, Drchco, Emd I/C, Emergency I/C, En I/C, En Lw Incharge, En Rch Incharge, Eye I/C, Eye Incharge, Facility Incharge, Female Ward I/C, Female Ward Incharge, Fw Nurse I/C, General Ward Incharge, General Wards Incharge, H/Pharm, Hdo Incharge, Head Incharge Maternity & Gyn, Head Icharge Medical, Head Incharge Laboratory, Head Incharge Pharmacy, Head Incharge Radiology, Head Of Emergency Department, Head Of Laboratory Department, Head Of Medical Department, Head Of Obs & Gyn Department, Head Of Paediatric Department, Head Of Pharmacy Unit, Head Of Quality Improvement Unit, Head Of Radiology Services, Head Of Rehabilitation Services, Head Of Surgical Department, Head Of Surgical Specialist, Health Assistant I/C, Hf. Incharge, Hims Fp, Hlab. Incharge, Hod Dental, Hod Engineer, Hod Eye, Hod Gyn/Obs, Hod Int.Medicine, Hod Internal Medicine, Hod Medical Department, Hod Obgy, Hod Opd, Hod Ophthalmology, Hod Ped Ward, Hod Pediatric, Hod Pharmacy, Hod Physiotherapy, Hod Qi, Hod Radiology, Hod Surg Ward, Hod Surgeon, Hod Surgery, Hod Surgical, Hod X-Ray, Hospital Laboratory In Charge, Hospital Laboratory Manager, Hospital Pharmacist In Charge, In Patient Incharge, Incharge Lab, Incharge Opd, Incharge Pharmacy, Incharge Laboratory, Incharge Anaesthesia, Incharge Ctc, Incharge Data, Incharge Female Ward, Incharge Lab, Incharge Male Ward, Incharge Nhif, Incharge Opd, Incharge Oth, Incharge Pharmacy, Incharge Radiology, Incharge Rch Ano, Inpatient Incharge, Ipd Incharge, Ipd Ass. Nurse Officer Incharge, Ipd I/C, Ipd Inchare (Ano), Ipd Inchare (En), Ipd Incharge, Ipd Incharge-Amo, Ipd Incharge-Co, Ipd Nurse Incharge, Ipd Nursing Incharge, Ipdm -Nurse Incharge-Female /Male Ward, Ipdm -Nurse Incharge-Female /Maleward, Ipdm -Nurse Incharge-Female Ward, Ipdm -Nurse Incharge-Female/Male Ward, L/W I/C, Lab I/C, Lab Incharge, Lab Assistant Incharge, Lab Inc, Lab Inch Ward I/C, Lab Incharge -Lt, Lab Incharge (Lt), Lab Incharge-H\A, Lab Incharge-Lab Assitant, Lab Incharge-Lab Attendant, Lab Incharge-Matt, Lab Manager, Lab Tech I/C, Lab.Tech-Incharge, Labor Word I/C, Laboratory Incharge, Laboratory I/C, Laboratory Tech Incharge, Labour And Rch Incharge -En, Labour I/C, Labour Incharge (Ano), Labour Incharge (En), Labour Incharge-Rn, Labour Ward I/C, Labour Ward Incharge, Labour Ward Incharge (Ano), Labour Ward Nurse Incharge, Lw I/C, Lw Nurse Incharge, Major Ot Ass. Nurse Officer Incharge, Malaria Focal Person, Male Ward I/C, Male Ward Incharge, Male Ward Incharge (En), Maternity Incharge, Maternity Incharge Mo And En, Mat Ward Nurse Ic, Maternity Nurse Incharge, Maternity Block Incharge, Maternity Dr Incharge, Maternity I/C, Maternity Nurse Incharge, Maternity Ward Ass. Nurse Incharge, Maternity Ward I/C, Maternity Ward Incharge, Maternity Ward Nurse Incharge, Medical Attendant In Charge (Reception), Medical Attendant In Charge (Dispensing), Medical Attendant In Charge (Ipd), Medical Attendant In Charge (Opd), Medical Attendant In Charge (Reception), Medical Officer Incharge (Co), Mmw Nurse Ic, Mortuary Depart Incharge, Mortuary Incharge, Msw Nurse I/C, Mtuh A Focal, Nhif Incharge, Nurse I/C Labour Ward, Nurse Incharge (Labour ), Nurse In Charge Female Ward, Nurse In Charge Pediatric Ward, Nurse In Charged Lw, Nurse Incharge Lw, Nurse Incharge Ctc, Nurse Incharge Lw, Nurse Incharge Male Ward, Nurse Incharge Opd, Nurse Incharge Rch, Nurse Incharge Ctc, Nurse Incharge Female Ward, Nurse Incharge Grade 2, Nurse Incharge Hmis, Nurse Incharge Ipd, Nurse Incharge Lishe, Nurse Incharge Male Ward, Nurse Incharge Op Theatre, Nurse Incharge Opd, Nurse Incharge Oth, Nurse Incharge Pediatric Ward, Nurse Incharge Pharmacy, Nurse Incharge Post C/S, Nurse Incharge Rch, Nurse Incharge Reception, Nurse Incharge Surgical Ward, Nurse Incharge Tb, Nurse Incharge ( Opd ), Nurse Incharge ( Rch ), Nurse Incharge ( Ward ), Nurse Incharge (Anc), Nurse Incharge (Ano)- Rch, Nurse Incharge (Ano)-Labour Ward, Nurse Incharge (Ctc), Nurse Incharge (Labour And Rch), Nurse Incharge (Labour Ward), Nurse Incharge (Labour), Nurse Incharge (Opd), Nurse Incharge (Patron), Nurse Incharge (Rch), Nurse Incharge (Rchc), Nurse Incharge (Surgery Ward), Nurse Incharge (Theater), Nurse Incharge (Ward, Nurse Incharge (Ward) Rn, Nurse Incharge (Ward3), Nurse Incharge (Ward4), Nurse Incharge Ant Natalward, Nurse Incharge Bima Block, Nurse Incharge Ctc, Nurse Incharge Eye Department, Nurse Incharge Female Ward, Nurse Incharge General Ward, Nurse Incharge Hdu, Nurse Incharge Icu, Nurse Incharge Ipd, Nurse Incharge Labour, Nurse Incharge Labour Ward, Nurse Incharge Lw, Nurse Incharge Male, Nurse Incharge Mart Wing, Nurse Incharge Maternity Block, Nurse Incharge Mat Ward, Nurse Incharge Maty, Nurse Incharge Medical Female, Nurse Incharge Medical Male, Nurse Incharge Mw, Nurse Incharge Opd, Nurse Incharge Ot, Nurse Incharge Pediatric Ward, Nurse Incharge Pediatric, Nurse Incharge Rch, Nurse Incharge Rchs, Nurse Incharge Rn, Nurse Incharge Surgical Female, Nurse Incharge Surgical Male, Nurse Incharge Theater, Nurse Incharge- Rch, Nurse Incharge- Rch (Ano), Nurse Incharge- Rch (En), Nurse Incharge-En, Nurse Incharge-Matt, Nurse Incharge-Opd, Nurse Incharge-Rn, Nurse Incharge-Theatre Depert., Nurse Incharge(Female Ward), Nurse Incharge(Male Ward), Nurse Incharge(Mat Ward), Nurse Incharge(Maternity Ward), Nurse Incharge(Matron), Nurse Incharge(Opd), Nurse Incharge(Ot), Nurse Incharge(Oth), Nurse Incharge(Paediatric Ward), Nurse Incharge(Rch), Nurse Incharge(Rchc), Nurse Incharge(Ward 1), Nurse Incharge(Ward 2), Nurse Incharge(Ward), Nurse Incharge(Ward)-Female Med-Surgical, Nurse Incharge(Ward)-Female Medical, Nurse Incharge(Ward)-Labour Ward, Nurse Incharge(Ward)-Male, Nurse Incharge(Ward)-Male Med-Surgical, Nurse Incharge(Ward)-Male Medical, Nurse Incharge(Ward)-Male Surgical, Nurse Incharge(Ward)-Maternity, Nurse Incharge(Ward)-Obs & Gyn, Nurse Incharge(Ward)-Padiatrict, Nurse Incharge(Ward)-Pediatric, Nurse Incharge(Ward)-Postnatal, Nurse Incharge(Ward)F, Nurse Incharge(Ward)M, Nurse Incharge(Ward)Mat, Nurse Incharge(Ward)Opd, Nurse Incharge(Ward)Pw, Nurse Ipd Incharge, Nurse Labour Ward Incharge, Nurse Officer Incharge (Opd/ L.Ward), Nurse Rch Incharge, Nurse Rch/Labour Ward Incharge, Nurse Rch/Opd Ward Incharge, Nursing Incharge (Ctc), Nursing Incharge (Ipd), Nursing Incharge (Labor ), Nursing Incharge (Labor Ward), Nursing Incharge (Maternity), Nursing Incharge (Opd), Nursing Incharge (Rch), Nursing Officer Incharge -En, Nursing Officer Incharge -Rn, Nursing Officer Incharge (Ano), Obs & Gyn Incharge, Opd Incharge, Opd I/C, Opd Incharge, Opd Nurse Incharge, Opd And Ipd Incharge-Mo, Opd Ass. Nurse Officer Incharge, Opd Co Incharge, Opd Dr Incharge, Opd I/C, Opd Incharge, Opd Incharge ( Ano), Opd Incharge (Co), Opd Incharge- Co, Opd Incharge- Mo, Opd Incharge-Co, Opd Incharge-Mo, Opd Inchargeand Rch Incharge, Opd Nurse I/C, Opd Nurse Ic, Opd Nurse Incharge, Opd Nursing Incharge, Opd Ward I/C, Opdi/C, Opdm-(Clinical Assistant) Incharge, Opdm-Addo Incharge, Opdm-Clinical Officer Incharge, Opdm-Nurse Incharge, Operating Theatre Incharge, Ot I/C, Ot Nurse Ic, Pae W Nurse I/C, Paediatric I/C, Paediatric Nurse Incharge, Paediatric Ward I/C, Paediatric Incharge, Pediatric Ward I/C, Pharmacy Incharge-, Pharmacy Incharge-Matt, Pharmacy Incharge, Pharm I/C, Pharm Incharge, Pharmaceutical Tech Incharge, Pharmacist Incharge, Pharmacy I/C, Pharmacy Incharge, Pharmacy Incharge-Matt, Pharmacy Incharge-Co, Post Nw Nurse I/C, Qi Focal, Qifp, Qit I/C, Radiologist I/C, Radiology I/C, Radiology Incharge-Radiologist, Rch Incharge, Rch Incharge (Ano), Rch And Labor Incharge-En, Rch And Labour Incharge-Rn, Rch I/C, Rch Incharge (En), Rch Incharge (Ano), Rch Incharge Ass. Nurse Officer Incharge, Rch Incharge-En, Rch Incharge-Mcha, Rch Incharge-Rn, Rch Incharge( Ano), Rch Incharge, Rch Nurse Incharge, Rch/Labour Nurse Incharge, Rchc Nurse I/C, Rchcs Ward I/C, Rchi/C, Rhc Incharge, Rn Ctc Incharge, Rn Facility Incharge, Rn Lw Incharge, Rn Opd Incharge, Rn Rch Incharge, Tb Focal (Ano), Tb Focal (Ma), Th Incharge, Theater In Charge, Theater Incharge, Theatre I/C, Theatre Icharge (Ano), Theatre Incharge, Theatre Incharge (Ano), Theatre Incharge (En), Theatre Incharge (Ma), Tivo, Town Environment Officer, Town Laboratory Technician, Tqi Focal Person, Traditional& Alt Medicine, Trchco, Ultrasound Incharge, Ward 1 I/C, Ward 10 I/C, Ward 1 Female I/C, Ward 2 I/C, Ward 2 Male I/C, Ward 3 Chw I/C, Ward 3 I/C, Ward 4 I/C, Ward 4 Maty I/C, Ward 5 I/C, Ward 6 I/C, Ward 7 I/C, Ward 8 I/C, Ward 9 I/C, Ward In Charge, X-Ray I/C |
| **Healthcare Provider** | Occupational Therapy, Amo, Anaesthetist, Anaethetist, Ano, Antenatal Ward, Ass Noi, Assistant Medical Officer, Assistant Nursing Officer, Asst Nursing Officer, C/A, C/O, Clinical Assistant, Clinical Officer, Clinical Service Coordinator, Clinical Services Co, Clinical Tracker (Ctc), Co, Csc, Csco, Ctc, Ctc Unit, Dental, Dental Clinic, En, Environmental Officer, Environment Health, Environmental, Epidemiologist, Eye, Eye Clinic, Eye Depart., Eye Department, Female Medical Ward, Female Ward, Female Ward (En), Grade One, Gyn Ward, Gyne Ward Dr, Health Officer, Hmis, Ho, Hospital Nursing Officer, Hospital Pharmacist, Hospital Social Welfare, Hpharm, Hqi, Hswo, Infusion, Insurance, Ipc, Ipd, Ipd (En), Ipd [ Female Ward], Ipd [ Pediatric Ward ], Ipd [Male Ward ], Ipd (Ano), Ipd Female, Ipd Male, Ipd Md, Ipd Nurse, Ipdm, L/Manager, Lab, Lab Assistant, Lab Tech, Lab Technician, Labor Ward, Laboratory, Labour, Labour Ward, Labr Tech., Lw, Major Theatre, Malaria Co, Male Medical Ward, Male Surgical Ward, Male Ward, Maternity Ward, Md, Md Maternity Ward, Medical Officer, Midwifery Unit, Ncd Co, Nhif, Nhif Co, Nhifco, Nicu Ward, Nurse, Nurse Labour Warb, Nursing Unit, Nutrition, Nutrition Officer, Nutritional Officer, Nutritionist, Obs N Gyne, Obs & Gyn, Obstetric And Gynaecology Ward, Obstetric/Gynaecology Ward, Obsy, Opd, Opd Bima, Opd Dr, Opd Emergency, Opd M, Opd Md, Opd Nurse, Opdm, Ophthalmia, Paediatric, Paediatric Ward, Parm, Parmacyst, Pediatric Nursing, Paediatric Ward, Pediatric, Pharm, Pharm Co (Pht), Pharm Tec, Pharm Tech, Pharmacist, Pharmacy, Pharm Assistant, Pharm Asisty, Pharmtec, Physiotherapy, Postnatal Ward, Psychiatric Unit, Qi, Qif, Quality Improvement Officer, R/N, Radiocrapher, Radiographer, Radiology, Radiology Department, Radiology Unit, Rch, Rn, School Health Co, So, Social, Social Welfare, Sow, Surgical Female, Surgery Male Ward, Swfo, Swo, Tb Clinic, Tb Co (Co ), Tb Co (En), Tb Co (Ma), Tb Unit, Tb/& Leprosy, Tb/Hiv Coordinator, Tb Leprosy, Tb&Liprose Co, Theater, Theatre, Town Nutrition Officer, Town Pharm, Town Pharmacist, Town Social Welfare Officer, Uss, Xray |
| **MOI** | Clinical Officer Incharge, HF Incharge, (Amo) Incharge, (Dental Officer)Incharge, (Incharge) Ass.Nurse Officer, Amo Incharge, Assistant Medical Officer Incharge, Assistant Clinical Officer I/C, Assistant Medical Officer Incharge, Asst Medical Officer Incharge, Ca I/C, Clinical Assistant Incharge, Clinical Officer Incharge, Clinician Incharge, Co I/C, Co Incharge, Coi/C, District Medical Officer, Dr Incharge, Dr. Incharge, En Facility I/C, Facility Incharge, Facility I/C, Facility Incharge (Aco), Facility I/C, Facility Incharge (Ano), Facility Incharge (Co), Facility Incharge (En), Facility Incharge (No), Facility Incharge (Nurse), Facility Incharge Co, Facility Incharge(Nurse), Health Facility Incharge, Health Facility Incharge (En), Health Facility Incharge (Ano ), Health Facility Incharge (Co), Health Facility Incharge (No), Health Facility Incharge En, Health Facility Incharge (Mo), Health Facility Incharge, Hf Incharge, Hf Incharges, In Charge, Incharge, Medical Officer Incharge, Medical Officer Incharge -Mo, Medical Officer Incharge (Amo), Medical Officer Incharge (Md), Medical Officer Incharge- Amo, Medical Officer Incharge- Mo, Medical Officer Incharge-Ca, Medical Officer Incharge-Co, Medical Officer Incharge/Ceo, Medical Officer Inchargeopdm, Mo I/C, Moi, Town Medical Incharge |
| **NOI** | Facility Incharge Nurse, Nurse Incharge, Ass Nursing Officer I/C (Matron), Ass. Nurse Officer, Ass. Nurse Officer Incharge, Ass. Nursing Officer Incharge, Assistant Nursing Officer (Matron), Assistant Nursing Officer I/C, Assistant Nursing Officer Incharge, Assistant Nursing Officer Incharge, Asst.Nursing Officer Incharge, Clinical Officer Incharge, District Nursing Officer, Facility Incharge Nurse, Matron, Matron (Ano), Matron (En), Matron (Enrolled Nurse), Matron/Patron, Matrone, No I/C, Noi, Noi/C, Nurse Incharge, Nurse In Charge, Nurse Officer Incharge, Nurse Officer Incharge And Labour-Rn, Nursing Incharge, Nursing Officer Incharge, Nursing Officer Incharge (Patron), Nursing Officer Incharge-Matron, Nursing Officer Incharge-Patron, Patron, Patron (Ano), Patrone, Town Nursing Officer |
| **Other** | E, IDSR, LBW, OS, TACC, VMMC |

**Table B. Regional Health Management Team Results**

|  | **Total** | **Women** | **Men** | **Unknown** | ***Ratios (Men: Women)*** |
| --- | --- | --- | --- | --- | --- |
| **Overall Gender** | | | | | |
|  | 370 | 146 | 220 | 4 | ***1:0.7*** |
|  | ***%*** | *39.5%* | *59.5%* | *1.1%* |  |
| **Position** | | | | | |
| **Regional Medical Officer** | 18 | 2 | 16 | -- | ***1:0.1*** |
|  | ***%*** | *11.1%* | *88.9%* | *--* |  |
| **Regional Nursing Officer** | 18 | 14 | 4 | -- | ***1:3.5*** |
|  | ***%*** | *77.8%* | *22.2%* | *--* |  |
| **Health Secretary** | 18 | 12 | 6 | -- | ***1:2*** |
|  | ***%*** | *66.7%* | *33.3%* | *--* |  |
| **Other** | 316 | 118 | 194 | 4 | ***1:0.6*** |
|  | ***%*** | *37.3%* | *61.4%* | *1.3%* |  |
| **Region** | | | | | |
| **ARUSHA** | 22 | 12 | 10 | -- | ***1:1.2*** |
|  | ***%*** | *54.5%* | *45.5%* | *--* |  |
| **DAR ES SALAAM** | 25 | 12 | 13 | -- | ***1:0.9*** |
|  | ***%*** | *48.0%* | *52.0%* | *--* |  |
| **DODOMA** | 24 | 12 | 12 | -- | ***1:1*** |
|  | ***%*** | *50.0%* | *50.0%* | *--* |  |
| **IRINGA** | 23 | 7 | 13 | 3 | ***1:0.5*** |
|  | ***%*** | *30.4%* | *56.5%* | *23.1%* |  |
| **KAGERA** | 19 | 7 | 12 | -- | ***1:0.6*** |
|  | ***%*** | *36.8%* | *63.2%* | *--* |  |
| **KILIMANJARO** | 15 | 7 | 7 | 1 | ***1:1*** |
|  | ***%*** | *46.7%* | *46.7%* | *14.3%* |  |
| **LINDI** | 21 | 7 | 14 | -- | ***1:0.5*** |
|  | ***%*** | *33.3%* | *66.7%* | *--* |  |
| **MARA** | 19 | 6 | 13 | -- | ***1:0.5*** |
|  | ***%*** | *31.6%* | *68.4%* | *--* |  |
| **MBEYA** | 18 | 10 | 8 | -- | ***1:1.3*** |
|  | ***%*** | *55.6%* | *44.4%* | *--* |  |
| **MTWARA** | 13 | 4 | 9 | -- | ***1:0.4*** |
|  | ***%*** | *30.8%* | *69.2%* | *--* |  |
| **MWANZA** | 27 | 11 | 16 | -- | ***1:0.7*** |
|  | ***%*** | *40.7%* | *59.3%* | *--* |  |
| **NJOMBE** | 24 | 11 | 13 | -- | ***1:0.8*** |
|  | ***%*** | *45.8%* | *54.2%* | *--* |  |
| **PWANI REGION** | 23 | 8 | 15 | -- | ***1:0.5*** |
|  | ***%*** | *34.8%* | *65.2%* | *--* |  |
| **RUKWA** | 19 | 5 | 14 | -- | ***1:0.4*** |
|  | ***%*** | *26.3%* | *73.7%* | *--* |  |
| **SHINYANGA** | 25 | 11 | 14 | -- | ***1:0.8*** |
|  | ***%*** | *44.0%* | *56.0%* | *--* |  |
| **SONGWE** | 15 | 4 | 11 | -- | ***1:0.4*** |
|  | ***%*** | *26.7%* | *73.3%* | *--* |  |
| **TABORA** | 20 | 6 | 14 | -- | ***1:0.4*** |
|  | ***%*** | *30.0%* | *70.0%* | *--* |  |
| **TANGA** | 18 | 6 | 12 | -- | ***1:0.5*** |
|  | ***%*** | *33.3%* | *66.7%* | *--* |  |

**Table C. Council Health Management Team Results**

|  | **Total** | **Women** | **Men** | **Unknown** | ***Ratios (Men: Women)*** |
| --- | --- | --- | --- | --- | --- |
| **Overall Gender** | | | | | |
|  | 2620 | 1087 | 1496 | 37 | ***1:0.7*** |
|  | ***%*** | *41.5%* | *57.1%* | *1.4%* |  |
| **Position** | | | | | |
| **District Medical Officer** | 125 | 22 | 103 | -- | ***1:0.2*** |
|  | ***%*** | *17.6%* | *82.4%* | *--* |  |
| **District Nursing Officer** | 126 | 95 | 31 | -- | ***1:3.1*** |
|  | ***%*** | *75.4%* | *24.6%* | *--* |  |
| **Health Secretary** | 125 | 56 | 67 | 2 | ***1:0.8*** |
|  | ***%*** | *44.8%* | *53.6%* | *--* |  |
| **Other** | 2244 | 914 | 1295 | 35 | ***1:0.7*** |
|  | ***%*** | *40.7%* | *57.7%* | *1.6%* |  |
| **Region (n=number of council teams)** | | | | | |
| **ARUSHA (n=7)** | 156 | 75 | 80 | 1 | ***1:0.9*** |
|  | ***%*** | *48.1%* | *51.3%* | *0.6%* |  |
| **DAR ES SALAAM (n=5)** | 123 | 72 | 51 | -- | ***1:1.4*** |
|  | ***%*** | *58.5%* | *41.5%* | *--* |  |
| **DODOMA (n=9)** | 201 | 75 | 99 | 27 | ***1:0.8*** |
|  | ***%*** | *37.3%* | *49.3%* | *13.4%* |  |
| **IRINGA (n=5)** | 98 | 46 | 51 | 1 | ***1:0.9*** |
|  | ***%*** | *46.9%* | *52.0%* | *1.0%* |  |
| **KAGERA (n=8)** | 152 | 54 | 98 | -- | ***1:0.6*** |
|  | ***%*** | *35.5%* | *64.5%* | *--* |  |
| **KILIMANJARO (n=7)** | 159 | 76 | 82 | 1 | ***1:0.9*** |
|  | ***%*** | *47.8%* | *51.6%* | *1.3%* |  |
| **LINDI (n=5)** | 108 | 41 | 67 | -- | ***1:0.6*** |
|  | ***%*** | *38.0%* | *62.0%* | *--* |  |
| **MARA (n=7)** | 153 | 51 | 102 | -- | ***1:0.5*** |
|  | ***%*** | *33.3%* | *66.7%* | *--* |  |
| **MBEYA (n=7)** | 70 | 26 | 43 | 1 | ***1:0.6*** |
|  | ***%*** | *37.1%* | *61.4%* | *1.4%* |  |
| **MOROGORO (n=2)** | 43 | 12 | 31 | -- | ***1:0.4*** |
|  | ***%*** | *27.9%* | *72.1%* | *--* |  |
| **MTWARA (n=9)** | 139 | 51 | 88 | -- | ***1:0.6*** |
|  | ***%*** | *36.7%* | *63.3%* | *--* |  |
| **MWANZA (n=8)** | 227 | 101 | 121 | 5 | ***1:0.8*** |
|  | ***%*** | *44.5%* | *53.3%* | *2.2%* |  |
| **NJOMBE (n=4)** | 111 | 38 | 73 | -- | ***1:0.5*** |
|  | ***%*** | *34.2%* | *65.8%* | *--* |  |
| **PWANI (n=9)** | 190 | 88 | 102 | -- | ***1:0.9*** |
|  | ***%*** | *46.3%* | *53.7%* | *--* |  |
| **RUKWA (n=4)** | 92 | 37 | 55 | -- | ***1:0.7*** |
|  | ***%*** | *40.2%* | *59.8%* | *--* |  |
| **SHINYANGA (n=4)** | 92 | 34 | 58 | -- | ***1:0.6*** |
|  | ***%*** | *37.0%* | *63.0%* | *--* |  |
| **SIMIYU (n=2)** | 37 | 18 | 18 | 1 | ***1:1*** |
|  | ***%*** | *48.6%* | *48.6%* | *2.7%* |  |
| **SONGWE (n=6)** | 98 | 40 | 58 | -- | ***1:0.7*** |
|  | ***%*** | *40.8%* | *59.2%* | *--* |  |
| **TABORA (n=6)** | 141 | 66 | 75 | -- | ***1:0.9*** |
|  | ***%*** | *46.8%* | *53.2%* | *--* |  |
| **TANGA (n=11)** | 230 | 86 | 144 | -- | ***1:0.6*** |
|  | ***%*** | *37.4%* | *62.6%* | *--* |  |

**Table D. Health Facility Management Team Results**

|  | **Total** | **Women** | **Men** | **Unknown** |  | ***Ratios (Men: Women)*** |
| --- | --- | --- | --- | --- | --- | --- |
| **Overall Gender** | | | | | | |
|  | **6130** | **2998** | **3072** | **60** | **1.0** | ***1:1*** |
|  | ***%*** | ***48.9%*** | ***50.1%*** | ***1.0%*** |  |  |
| **Position** | | | | | | |
| **Medical Officer In Charge** | **2091** | **623** | **1451** | **17** | **0.4** | ***1:0.4*** |
|  | ***%*** | ***29.8%*** | ***69.4%*** | ***--*** |  |  |
| **Nursing Officer In Charge** | **1407** | **927** | **469** | **11** | **2.0** | ***1:2*** |
|  | ***%*** | ***65.9%*** | ***33.3%*** | ***--*** |  |  |
| **Health Secretary** | **47** | **30** | **16** | **1** | **1.9** | ***1:1.9*** |
|  | ***%*** | ***63.8%*** | ***34.0%*** | ***--*** |  |  |
| **Health Section In Charge** | **1643** | **975** | **641** | **27** | **1.5** | ***1:1.5*** |
|  | ***%*** | ***46.6%*** | ***30.7%*** | ***--*** |  |  |
| **Healthcare Provider** | **815** | **393** | **419** | **3** | **0.9** | ***1:0.9*** |
|  | ***%*** | ***27.9%*** | ***29.8%*** | ***--*** |  |  |
| **Admin In Charge** | **19** | **9** | **10** | **0** | **0.9** | ***1:0.9*** |
|  | ***%*** | ***19.1%*** | ***21.3%*** | ***--*** |  |  |
| **Admin Support** | **98** | **33** | **64** | **1** | **0.5** | ***1:0.5*** |
|  | ***%*** | ***70.2%*** | ***136.2%*** | ***--*** |  |  |
| **Other** | **10** | **8** | **2** | **0** | **4.0** | ***1:4*** |
|  | ***%*** | ***80.0%*** | ***20.0%*** | ***0.0%*** |  |  |
| **Facility Type** | | | | | | |
| **Dispensary (n=1793)** | **3663** | **1766** | **1868** | **29** | **0.9** | ***1:0.9*** |
|  | ***%*** | ***84.5%*** | ***89.3%*** | ***--*** |  |  |
| **Health Centre (n=263)** | **1394** | **701** | **670** | **23** | **1.0** | ***1:1*** |
|  | ***%*** | ***49.8%*** | ***47.6%*** | ***--*** |  |  |
| **District & District-Level Hospitals (n=43)** | **928** | **467** | **454** | **7** | **1.0** | ***1:1*** |
|  | ***%*** | ***993.6%*** | ***966.0%*** | ***--*** |  |  |
| **Regional & Regional-Level Hospitals (n=6)** | **145** | **64** | **80** | **1** | **0.8** | ***1:0.8*** |
|  | ***%*** | ***3.1%*** | ***3.8%*** | ***--*** |  |  |
| **Region (n=number of facilities)** | | | | | | |
| **DAR ES SALAAM (n=19)** | **229** | **132** | **94** | **3** | **1.4** | ***1:1.4*** |
|  | ***%*** | ***6.3%*** | ***4.5%*** | ***--*** |  |  |
| **DODOMA (n=2)** | **13** | **7** | **6** | **0** | **1.2** | ***1:1.2*** |
|  | ***%*** | ***0.5%*** | ***0.4%*** | ***--*** |  |  |
| **IRINGA (n=216)** | **569** | **319** | **245** | **5** | **1.3** | ***1:1.3*** |
|  | ***%*** | ***678.7%*** | ***521.3%*** | ***--*** |  |  |
| **KAGERA (n=10)** | **20** | **11** | **9** | **0** | **1.2** | ***1:1.2*** |
|  | ***%*** | ***0.5%*** | ***0.4%*** | ***--*** |  |  |
| **KILIMANJARO (n=161)** | **509** | **310** | **184** | **15** | **1.7** | ***1:1.7*** |
|  | ***%*** | ***14.8%*** | ***8.8%*** | ***--*** |  |  |
| **LINDI (n=143)** | **298** | **124** | **171** | **3** | **0.7** | ***1:0.7*** |
|  | ***%*** | ***5.9%*** | ***8.2%*** | ***--*** |  |  |
| **MARA (n=149)** | **522** | **214** | **291** | **17** | **0.7** | ***1:0.7*** |
|  | ***%*** | ***10.2%*** | ***13.9%*** | ***--*** |  |  |
| **MBEYA (n=26)** | **78** | **33** | **45** | **0** | **0.7** | ***1:0.7*** |
|  | ***%*** | ***1.6%*** | ***2.2%*** | ***--*** |  |  |
| **MOROGORO (n=30)** | **143** | **73** | **70** | **0** | **1.0** | ***1:1*** |
|  | ***%*** | ***5.2%*** | ***5.0%*** | ***--*** |  |  |
| **MWANZA (n=321)** | **728** | **345** | **382** | **1** | **0.9** | ***1:0.9*** |
|  | ***%*** | ***734.0%*** | ***812.8%*** | ***--*** |  |  |
| **NJOMBE (n=206)** | **378** | **192** | **186** | **0** | **1.0** | ***1:1*** |
|  | ***%*** | ***9.2%*** | ***8.9%*** | ***--*** |  |  |
| **PWANI (n=227)** | **797** | **444** | **352** | **1** | **1.3** | ***1:1.3*** |
|  | ***%*** | ***21.2%*** | ***16.8%*** | ***--*** |  |  |
| **RUKWA (n=216)** | **322** | **117** | **204** | **1** | **0.6** | ***1:0.6*** |
|  | ***%*** | ***5.6%*** | ***9.8%*** | ***--*** |  |  |
| **SHINYANGA (n=124)** | **257** | **111** | **146** | **0** | **0.8** | ***1:0.8*** |
|  | ***%*** | ***5.3%*** | ***7.0%*** | ***--*** |  |  |
| **SIMIYU (n=15)** | **70** | **34** | **35** | **1** | **1.0** | ***1:1*** |
|  | ***%*** | ***1.6%*** | ***1.7%*** | ***--*** |  |  |
| **SONGWE (n=197)** | **500** | **213** | **275** | **12** | **0.8** | ***1:0.8*** |
|  | ***%*** | ***10.2%*** | ***13.2%*** | ***--*** |  |  |
| **TABORA (n=209)** | **688** | **319** | **368** | **1** | **0.9** | ***1:0.9*** |
|  | ***%*** | ***15.3%*** | ***17.6%*** | ***--*** |  |  |
| **TANGA (n=9)** | **9** | **0** | **9** | **0** | **0.0** | ***1:0*** |
|  | ***%*** | ***0.0%*** | ***0.4%*** | ***--*** |  |  |
